# Local Modelling Techniques for Assessing Micro-Level Impacts of Risk Factors in Complex Data: Understanding Health and Socioeconomic Inequalities in Childhood Educational Attainments

**DOI:** 10.1371/journal.pone.0113592

**Published:** 2014-11-19

**Authors:** Shang-Ming Zhou, Ronan A. Lyons, Owen G. Bodger, Ann John, Huw Brunt, Kerina Jones, Mike B. Gravenor, Sinead Brophy

**Affiliations:** 1 Institute of Life Science, College of Medicine, Swansea University, Swansea, United Kingdom; 2 Public Health Wales, Temple of Peace and Health, Cathays Park, Cardiff, United Kingdom; Institute for Health & the Environment, United States of America

## Abstract

Although inequalities in health and socioeconomic status have an important influence on childhood educational performance, the interactions between these multiple factors relating to variation in educational outcomes at *micro-level* is unknown, and how to evaluate the many possible interactions of these factors is not well established. This paper aims to examine multi-dimensional deprivation factors and their impact on childhood educational outcomes at micro-level, focusing on geographic areas having widely different disparity patterns, in which each area is characterised by six deprivation domains (*Income*, *Health*, *Geographical Access to Services*, *Housing*, *Physical Environment*, and *Community Safety*). Traditional health statistical studies tend to use one global model to describe the whole population for *macro-analysis*. In this paper, we combine linked educational and deprivation data across small areas (median population of 1500), then use a *local modelling* technique, the Takagi-Sugeno fuzzy system, to predict area educational outcomes at ages 7 and 11. We define two new metrics, “*Micro-impact of Domain*” and “*Contribution of Domain*”, to quantify the variations of local impacts of multidimensional factors on educational outcomes across small areas. The two metrics highlight differing priorities. Our study reveals complex multi-way interactions between the deprivation domains, which could not be provided by traditional health statistical methods based on single global model. We demonstrate that although *Income* has an expected central role, all domains contribute, and in some areas *Health*, *Environment*, *Access to Services*, *Housing* and *Community Safety* each could be the dominant factor. Thus the relative importance of health and socioeconomic factors varies considerably for different areas, depending on the levels of each of the other factors, and therefore each component of deprivation must be considered as part of a wider system. Childhood educational achievement could benefit from policies and intervention strategies that are tailored to the local geographic areas' profiles.

## Introduction

Increasing evidence shows that childhood health and socioeconomic inequalities have strong impact on educational attainments [1]–[7]. It is often the case, however, that existing studies focus on the impact of *either* health *or* socioeconomic status, and research results are often obtained at a *macro-level*, whereas the subtle analysis of risk factor interactions at a *micro-level* across different subgroups is much less understood in epidemiological and public health studies. Such considerations might reveal more complex relationships and provide important insights for targeted policy development and intervention. However, currently few statistical analytics methods and modelling techniques can fulfil the tasks of conducting subtle analysis of risk factor interactions at the *micro-level* while maintaining the *macro-level* system performance.

This paper offers a local modelling technique, the Takagi-Sugeno (TS) fuzzy system [8] for characterising subtle relationships between independent variables (inputs) and the dependent variable (output) across different local data regions while constructing a global system model. As a popular modelling technique, TS fuzzy models have gained successful applications in many areas [9]–[11], but few studies have examined the roles of TS fuzzy models in characterising subtle relationships between inputs and output across local data regions. There are a number of challenges in applying TS fuzzy models to local characterisation of data space. First, the number of TS fuzzy rules (corresponding to local linear models) increases exponentially along with the growth of the number of independent variables (the *curse of dimensionality*). In order to tackle this challenge, Zhou et al proposed a method of constructing compact TS model [12], in which the redundant local linear models (LLMs) are removed, and only important ones are used in the final model. Second, currently there are no effective analytic tools for TS fuzzy models to quantitatively assess the impacts of input variables on output across different local data regions. To this end, this paper proposes two new metrics: One is called “*Micro-impact of Domain*” (*MiD*) to assess the expected impact of changes in an input variable on outcome at a micro-level by fusing the coefficients of dominated LLM and all other LLMs in a given data sub-region. We further propose a second metric: “*Contribution of Domain*” (CoD) to assess the gross contribution of each domain at the corresponding sub-region.

Using the two new metrics we aim to provide new insights into the complex relationships between health and socioeconomic status, and educational attainments at the small area level based on data linked across routine databases. The significance of research on area-based effects lies in the need to focus public health and socioeconomic promotion initiatives on the broader characteristics of places where disadvantaged people live [13]–[20]. Understanding how drivers such as poor health, poor housing, poor local environment, unstable communities and poor public service support, interact to create a cycle of decline and underachievement is vital to identify the most appropriate policy responses.

## Materials and Methods

### Indices of childhood deprivation

We performed a cross-sectional study linking routine data from the Welsh Child Index for Multiple Deprivation (WCIMD) [21] with data from the UK National Pupil Database (NPD) [22]. In the UK, a considerable amount of data is produced at the so-called Lower Super Output Area (LSOA) level. LSOAs have a median population of approximately 1500 people, and were created by the UK Office for National Statistics as permanent census geographies, taking into account population size, proximity, and social homogeneity [23].

A variety of indices of area level deprivation have been created for LSOAs, by governments in different parts of the UK. The WCIMD was designed specifically for childhood policy development as part of the Neighbourhood Statistics programme in England and Wales, UK [21]. The WCIMD is based on seven separate domains of deprivation: *Income*, *Health*, *Access (Geographic) to Services*, *Housing*, *Physical Environment*, *Community Safety* and *Education* (*including skills and training*) at the LSOA level [21]. The indicators for each domain are based on comprehensive and robust criteria, and are regularly updated. The 2008 recently revised version of WCIMD [24] [25] is calculated for each of the 1896 LSOAs across Wales. Each domain of the index is scored on a scale of 0 to 100 (highest level of deprivation) and is itself a composite variable. For example: *Income* captures the proportion of children living in households with income below a defined threshold or claiming benefits relating to low incomes. *Health* measures the degree to which children are deprived of good health, as determined by the area prevalence of limiting long-term illness and low birth weight. *Housing* captures deprivation though lack of central heating and indicators of overcrowding. *Environment* represents physical environmental risk factors that may impact quality of life, including air quality, air emissions, flood risk and proximity to waste disposal and industrial sites. *Access* represents deprivation resulting from difficulties in accessing a range of necessary services (average travel time to primary care centres, schools, libraries, leisure facilities). *Community* represents safety, based on police recorded crime, numbers of youth and adult offenders and incidents of fire. Since our target outcome variable is educational achievement, which is itself a component of the seventh WCIMD domain, we excluded the *Education* component of WCIMD from our analysis.

Simple investigation of the data may reveal that some of the domains (e.g. *Access* and *Environment*) are not strongly associated with educational attainment. However, in practice, the indicators used to define the two deprivation indices can potentially have direct or indirect impact on educational performances. For example, long distances to schools and transport nodes could worsen educational performances for children from poor family backgrounds. The *Environment* indicators, such as air quality, can significantly affect child's health, then impact educational performance indirectly. This is particularly true for children with existing poor health conditions. Substantial evidence has shown the adverse effects of exposure to ambient air pollutants on the development of lung function, and aggravation of asthma [26]. These effects may only be apparent at certain local regions, and also in combination (interaction) with other deprivation domains. Such subtle local effects, that are missed at the global level and that may exist across and between all deprivation domains, are the motivation and target of our study.

### Educational under-attainment rates

**Figure 1:**
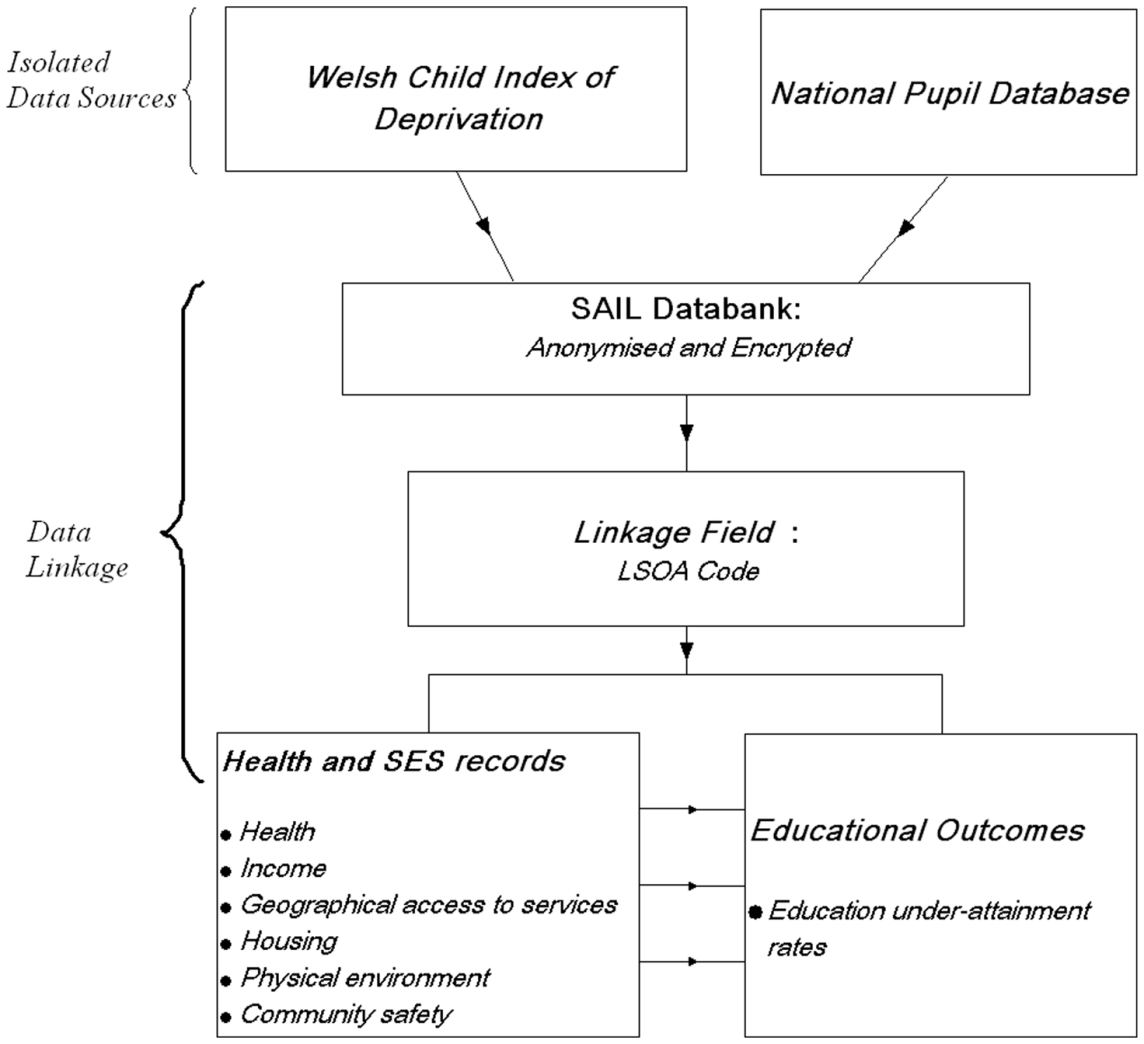
Linkage of data from Welsh Child Index of Multiple Deprivation with educational outcomes (under-attainment rate) at Lower Super Output Area level via Secure Anonymised Information Linkage (SAIL) databank.

The UK NPD [22] matches information from the Pupil Level Annual School Census (PLASC) with records on key stage (KS) educational attainment. We focused on educational attainment at KS1 (age 6–7) and KS2 (age 10–11). Categorisation is based on whether a pupil attains an expected level of attainment for all three core subjects: *Mathematics*, *Science*, and *English* or *Welsh* (depending on first language). Here, we defined the overall *education under-attainment rate* (EUR) at the LSOA level as a proportion, being the total number of children achieving lower than expected levels (KS1 and KS2) divided by the total number of child assessments made during the same period represented by the WCIMD indices. Education data at the LSOA level was collected from anonymised individual records, and linked to deprivation indices, using the *SAIL (Secure Anonymised Information Linkage)* databank [27] [28] (Figure 1).

### TS fuzzy modelling framework

This study is based on a local modelling technique, the TS fuzzy rule system [8] and a recently developed tool suited for detection of complex interactions in epidemiological data [12].

Different from the traditional health statistics for macro analyses, the TS fuzzy system decomposes the whole data space into individual regions, a local linear model (LLM) is fitted in every region, with the overall system output given as a global model which is obtained by fusing the submodels. Specifically, a TS fuzzy model is expressed as follows:
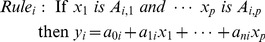
(1)where, 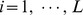, *y_i_* is the output variable of the *i*th rule, 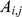 is a fuzzy set of the *j*th domain in the *i*th rule, and 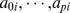 are consequent coefficients of the *i*th rule. An overall output *y* is produced by fusing together these *LLMs* 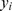 as
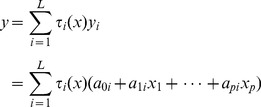
(2)where
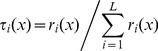
(3)is the normalized firing strength of the *i*th rule, and 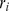 is usually defined by
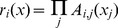
(4)in which the 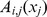
is the membership function of the fuzzy set 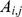. The overall fused system model (2) is also called the *global* model. The coefficients determine the size and direction of the effects in the local fuzzy region. For example, for the 6 deprivation domains, if each is categorized into regions “high” and “low”, there would be 64 ( = 2^6^) sub-regions representing all combinations of each domain. The overall system output is obtained by fusing the outputs of all these submodels. The output is a predicted (fitted) education under-attainment rate for each LSOA (geographic area).

In essence, we might say “*if all deprivation levels are ‘low’, then use Rule (LLM) 1*”. But in practice we wish to utilise our continuous data for deprivation, and might not be able to confidently classify each deprivation level as ‘low’. Therefore the specific LSOA prediction is obtained as a weighted average of *all* LLMs with the weights determined by how closely the specific LSOA fits into each sub-region characterised by fuzzy sets. The complexity of the model is reduced, by fitting local simple models to partitions of the data space, a *micro characterizations of input space* [29] [30]. In practice, one may tend to find a dominant rule for each sub-region. In this manner, TS rule modelling technique offers a novel way of revealing the subtle relationships between independent variables (inputs, the domains of deprivation) and dependent variable (output, the education under attainment rate) across very many small geographic regions.

Although the method is appealing, a cost is the “curse of dimensionality” whereby for many variables or sub-regions, there may be a large number of LLMs, which can lead to over-fitting and poor out-of-sample performance. Zhou et al have developed a method to identify a parsimonious set of rules that capture the key interactions between the variables whilst avoiding such over-fitting [12].

### Output metrics for the TS model

In this paper we propose two new metrics to quantitatively assess how the impacts of domain factors on outcome vary across these local regions at micro-level.

#### 1. MiD: micro-impact of domain

First note that in a TS fuzzy system, each LLM describes the relationship between domain factors and outcome in a certain data sub-region. The coefficients of these LLMs represent, for each deprivation domain, the rates and directions of expected change of the conditional mean of the outcome (e.g. education under-attainment rate) with respect to each deprivation domain. To make a prediction for a specific LSOA, the coefficients across all LLM are fused with the weights determined by how well the LSOA fits to each LLM data space region. These averaged coefficients are unique to each LSOA and give an indication of how the educational achievement would be expected to change given changes in deprivation scores. We denote this metric by the term “*Micro-impact of Domain*” (*MiD*) at the given sample point.

Specifically, given an LLM 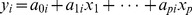, the slope coefficient vector *E* = (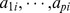)*^T^* is be normalized into a unitary vector:
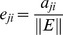
(5)Then given an input (e.g. vector of WCIMD deprivation scores) 
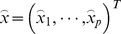
, the overall influence of the *j* ^th^ domain on output at the point 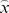 is defined as
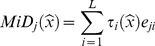
(6)Hence the *MiD* defines *the overall expected change in the output (e.g. education under-attainment rate)*, *on average*, *for a one-unit change in the j^th^ domain at the point* 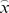 *, while holding all the other domains fixed*. It gives the size and direction of the effect that the *j^th^* domain is having on the output at the point 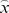. Note that *x* represents a general variable, while 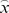 denotes the values of *x* at a specific point.

#### 2. CoD: contribution of domain

The MiD coefficient represents the slope of the relationship between deprivation and education, hence a standardised effect size for a given change in deprivation. In practice, domain scores for certain LSOAs may differ widely in magnitude, and potential changes in deprivation score via policy intervention may also differ widely, hence the absolute change in educational score needs to take this into account. The “*Contribution of Domain*” (CoD) is defined as the product of the MiD and the actual domain value.

Specifically, given an input sample, 
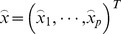
, the overall influence of the *j^th^* domain on 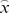 is defined as
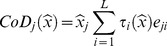
(7)


### Fitting the TS model

First, given the 1896 LSOA samples across Wales, a 9-fold cross validation scheme was used to evaluate the performance of the TS model. In each data partition, 1400 samples were used for training, 200 samples for validation, and 296 samples for testing. The 1400 training samples were used to fit the initial TS model with inputs representing the various indices of deprivation: *income* (*x_1_*), *health* (*x_2_*), *access to services* (*x_3_*), *housing* (*x_4_*), *physical environment* (*x_5_*), *community safety* (*x_6_*) and one output: *education educational under-attainment rate* (*y*). In this study, each of the deprivation domains was characterised into ‘low’ *non-deprived* scores and ‘high’ *highly deprived* categories. Since such classification has uncertainty associated with it, fuzzy sets were used to characterise these linguistic terms. First, the fuzzy c-means unsupervised clustering algorithm [31] was used to partition input space. By projecting the multi-dimensional prototypes on the input variable space [32], the membership functions of fuzzy sets were generated with the core cut-off points shown in the Table 1. In this way, this initial TS model consists of 64 rules representing all combinations of each domain in partitioning the input space. Then the validation data set was used to select the important rules and remove the redundant ones in the interest of constructing compact model using the *ω*-index of TS fuzzy rules proposed in [12]. The generalisation performance of the constructed compact TS model was evaluated by applying to the testing data set. Then the most compact TS model with good global prediction performance was used to analyse the micro-impacts of deprivation domains on educational under-attainment rate across different data sub-regions. All models were implemented in Matlab, and code is available upon request from the corresponding author.

**Table 1:** Core cut-off points for fuzzy sets to characterise “non-deprived” and “highly deprived” scores.

*Input variables*	*Non-deprived core scores*	*Highly deprived core scores*
Income	[0, 11.71]	[36.56, 100]
Health	[0, 13.14]	[35.2, 100]
Access	[0, 14.66]	[26.05, 100]
Housing	[0, 14.77]	[31.77, 100]
Environment	[0, 18.80]	[25.91, 100]
Community	[0, 12.24]	[36.63, 100]

### Ethics

The data used in this study was collected via national databases held in the *SAIL (Secure Anonymised Information Linkage)* databank [27] [28]. No ethical review was required because the SAIL databank holds the data which has been anonymised and granted with the permission of relevant Caldicott Guardian/Data Protection Officer [33], however, approval to proceed with the study was given by the Information Governance Review Panel [28].

## Results

**Figure 2:**
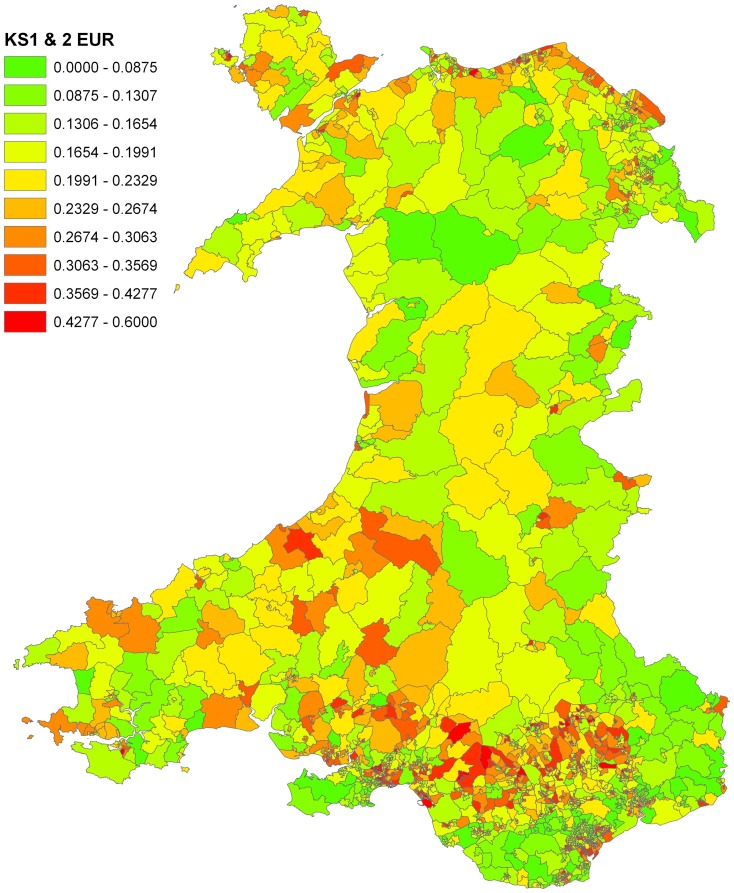
Educational under-attainment rates (EUR) in each LSOA across Wales.

There were 196,770 KS1 and KS2 childhood records for Wales during the relevant WCIMD period. The number of children with KS 1 or KS2 records in this study between 2005 and 2007 can be found in Table S1 of Data Supplement. Figure 2 depicts the educational under-attainment rate (EUR) across each of the 1896 LSOAs in Wales. The overall percentage failing to achieve the education target was 22.7%, with considerable geographical heterogeneity. The pair-wise linear correlation coefficients for all domains: *Income*, *Health*, *Access*, *Housing*, *Environment* and *Community*, can be found in Table S2 of Data Supplement. Fuzzy sets with the core cut-off points shown in the Table 1 were used to characterise the ‘low’ *non-deprived* scores and ‘high’ *highly deprived* categories. Uncertainty emerges for the areas whose deprivation scores lie between the cut-offs. For example, an LSOA with income deprivation score below 11.71 should belong, with high certainty, to the *non-deprived* category on *Income*, and one with deprivation score above 36.56 to the *highly deprived* category. In contrast an LSOA with income deprivation scores of 30 would have the membership of the *non-deprived* category with a “degree” (representing uncertainty) of 0.29 and at the same time, a degree of 0.84 belonging to the *highly-deprived* category. So in decision making about such areas, both high and low deprivation on *Income* (with their associated “*if-then*” rules) will contribute significantly, with the *weights* of each calculated from the degree of membership.

Using sub-regions of the data space based on the two sets of each of the 6 deprivation domains results in 64 rules (LLMs), and a risk of redundancy and over fitting of the data. Application of our recently developed algorithm [12] resulted in a compact, and parsimonious TS model. The 9 fold cross validation scheme showed an overall root-mean-squared-error (RMSE) of 0.0888 (95% confidence interval: [0.0845, 0.0931]). The best compact TS model had only 8 important rules (note that this is fewer than found in [12] as we are now using an updated version of the WIMCS data set). Taken together, these were sufficient to provide an accurate prediction of the education under-attainment rate in all LSOAs (generalization performance on testing data set with RMSE of 0.0842, see Figure S1 of Data Supplement). This means that the whole population of children attending the KS1 and KS2 across Wales can be grouped into 8 different clusters with similar characteristics in each cluster according to their combinations of deprivation domains of the WCIMD, and each cluster is dominated by an LLM.

**Figure 3:**
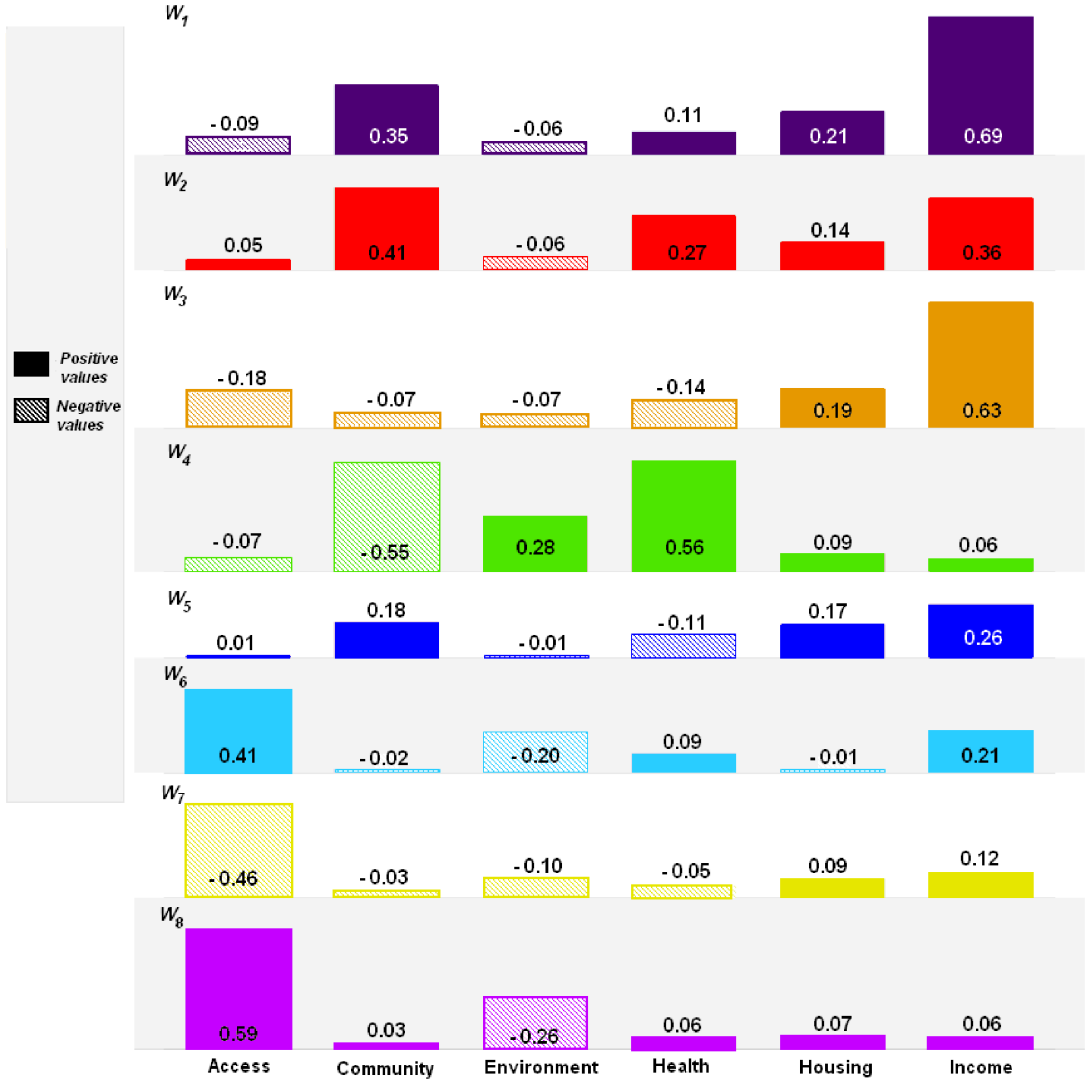
The coefficients of the eight local linear models in the parsimonious constructed compact TS system model. The rules are coded as *W_1_* to *W_8_* (see Table 2). Note that individual LSOAs have predicted values that are weighted averages of these 8 models, with the weights determined by the uncertainty of membership of the sub-regions.

**Figure 4:**
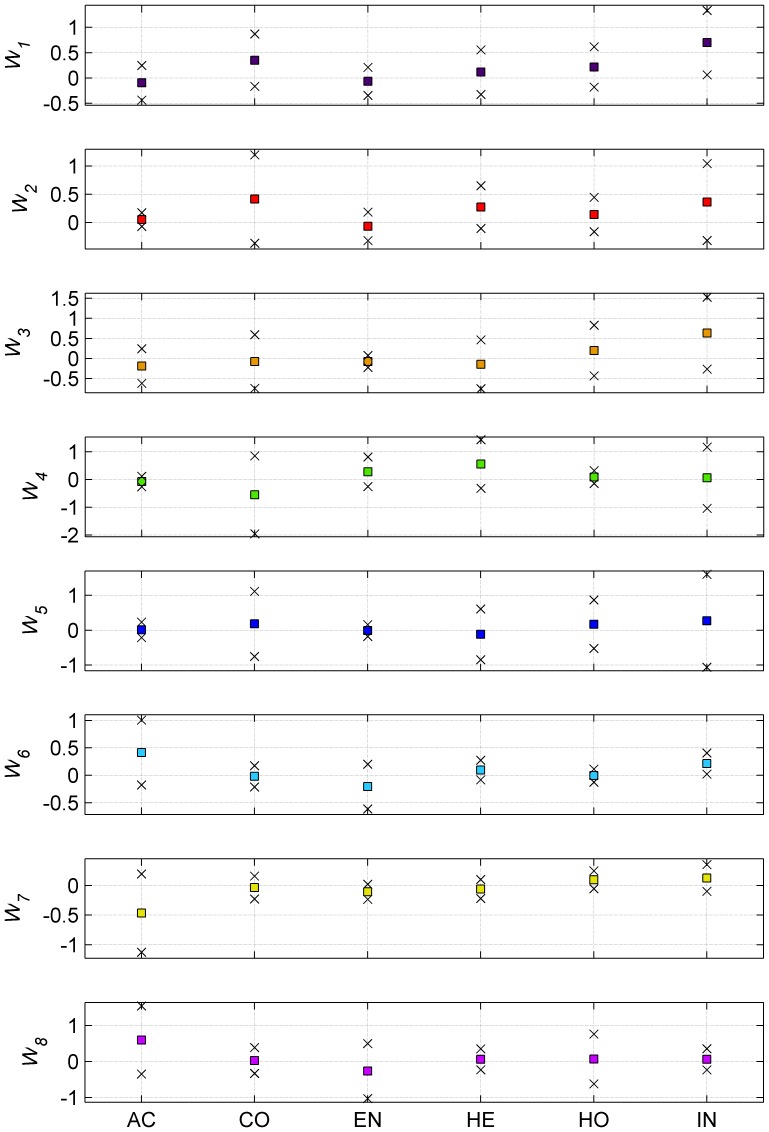
Properties of the coefficients of the eight selected “parsimonious” local linear models (LLMs). Green square represents point estimate, and “x” marks upper and lower 95% confidence intervals. (*IN- Income; HE- Health; AC- Access to Services; HO – Housing; EN – Physical Environment; CO- Community Safety*).

**Figure 5:**
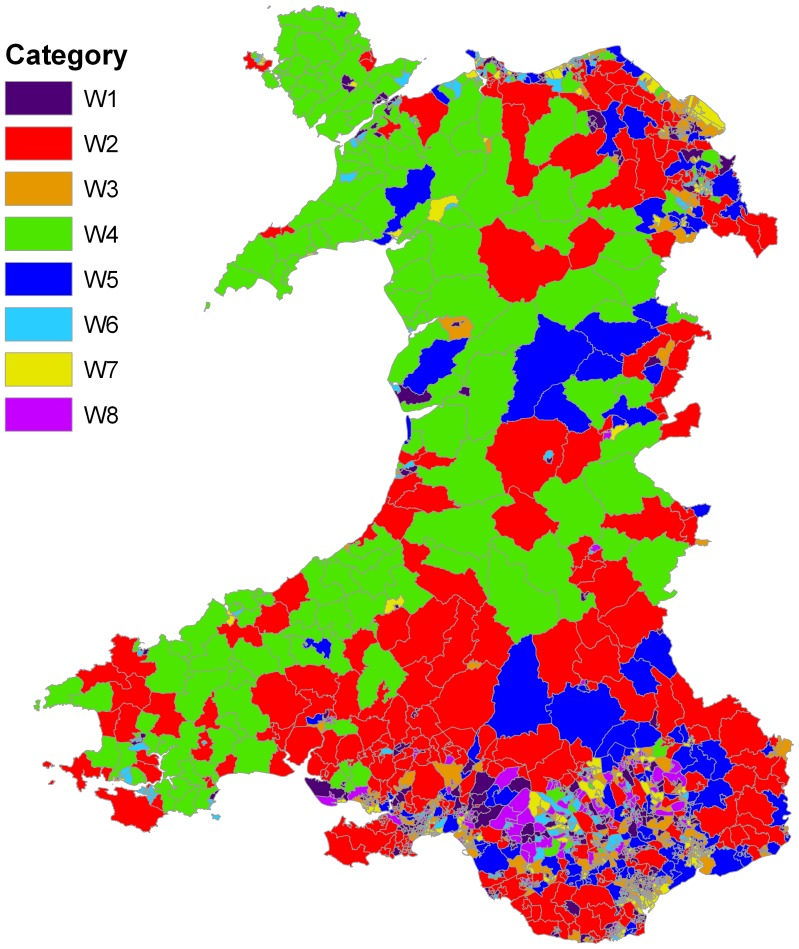
Map of Wales showing Lower Super Output Areas (LSOAs) coded according to the dominant rule at each LSOA that represents the relationship between deprivation domains and education under-attainment rate.

The input conditions for each rule are outlined in Table 2, and the coefficients of each of the 8 local linear models are given in Figure 3, while Figure 4 depicts the 95% confidence intervals (CIs) of these coefficients. The colours in Figure 3 are used to differentiate these LLMs and the corresponding clusters of LSOAs revealed in Figure 5. It can be seen that based on the given WCIMD training dataset, some coefficients of the LLMs have the negative values. These negative values represent the “unexpected” conclusion that education scores tend to *improve* with increased deprivation of corresponding domain, and tend to have wide confidence intervals associated with them. Hence although these trends are present in certain sub-regions of the data, evidence for such an effect is not very strong. The 95% CIs in Figure 4 show the range of plausible values that can act as estimates for the coefficients of each deprivation score in each LLM. *Income* domain in the LLMs *W_1_* and *W_6_* is clearly statistically significantly different from zero. The CIs for the domains of *Access* in *W_2_*, *Environment* in *W_3_*, *Health* in *W_6_*, and *Housing* in *W_7_* all also indicate statistical significance. We note that even small coefficients can make considerable contributions to the predicted education failure rate in local areas of high deprivation. This is because the expected education failure rate is obtained from the product of the coefficient and the deprivation score. This is part of the rationale for the choice of the CoD metric as a key output.

**Table 2:** The eight most important “*if-then*” input components of the rules (*W_1_∼W_8_*) for the final TS fuzzy model.

Rule ID	Access	Community	Environment	Health	Housing	Income
*W_1_*	-	-	-	-	-	-
*W_2_*	D	-	-	-	-	-
*W_3_*	-	-	D	-	-	-
*W_4_*	D	-	-	-	D	-
*W_5_*	D	-	D	-	-	-
*W_6_*	-	D	-	D	D	D
*W_7_*	-	D	D	D	D	D
*W_8_*	-	D	-	D	-	D

(These rules are selected from the 64 possible Local Linear Models according to our algorithm [12]. D = high deprivation, - = non-deprived).

As indicated above, each data sub-region has one dominating LLM while the rest of other LLMs play minor roles in prediction for the samples in this sub-region. Summarising the LLMs in the whole data space, Table 3 shows the values of mean, minimum and maximum of the LLMs' coefficients and corresponding 95% CIs. The Table 3 indicated that in overall, the *Income* and *Housing* domains are statistically significant, as might be expected, *Income* was the most influential domain.

**Table 3:** Summary of coefficients of the local linear models in the constructed TS system model.

*Variable*	*Baseline*	*Access score*	*Community score*	*Environment score*	*Health score*	*Housing score*	*Income score*
Minimum	7.09	−0.47	−0.55	−0.26	−0.14	−0.00	0.06
Maximum	30.88	0.60	0.42	0.28	0.56	0.22	0.7
Mean	16.31	0.03	0.04	−0.06	0.10	0.12	0.30
95%CI	[9.96, 22.66]	[−0.25, 0.31]	[−0.21, 0.29]	[−0.20, 0.07]	[−0.10, 0.29]	[0.06, 0.19]	[0.10, 0.51]

To further interpret these LLMs, the input conditions from Table 2 (the “*if*” component of the rules) can be combined with the coefficients from Figure 3 (the “*then*” component of the rules) to illustrate the extremely complicated interactions that emerge from the data mining. The effects of all individual deprivation domains were strongly dependent on the categories of all other domains in determining educational outcomes. This means that generalisations about the effect of any deprivation index to a local level could have poor performance if the other factors are not properly taken into account.

There are several outputs from the model that are of interest. First, a simple indicator that can be used to classify each LSOA is the “dominant rule”. Figure 5 shows the distribution of these 8 types of relationship across Wales revealed by the 8 LLMs in Figure 3 (colours used in these two Figures to represent the LLMs and corresponding LSOAs are consistent). Several interesting patterns emerge. The urban and rural areas of Wales are naturally marked by the size of the LSOA, with very small LSOAs found in the north and south coastal cities, as well as the South Wales industrial valleys. If we concentrate instead on the remaining rural areas, there remains a general north-west (NW) to south-east (SE) divide, with the NW characterised by rule 4 (green) in which the relationship between education under attainment and deprivation is strongly influenced by *Health*, with a contribution from *Environment* but a surprisingly small role for *Income*. While in the SE rule 2 dominates (red) in which *Income* plays a much more central role, along with *Community* and, to a lesser extent, *Health*.

**Figure 6:**
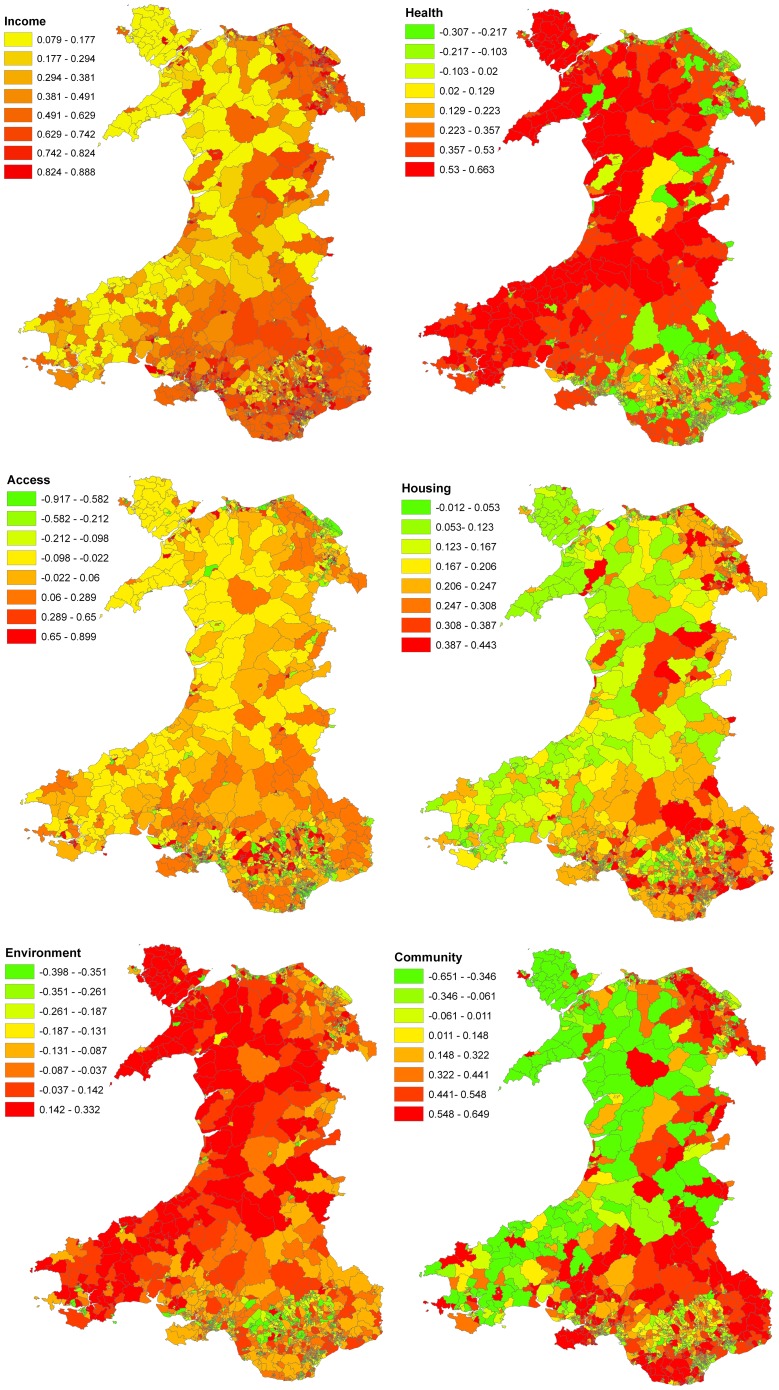
Variations in Micro-impact of Domain (MiD). Overall rates and directions of expected changes in education under attainment rate, for unit change in domain deprivation score.

**Figure 7:**
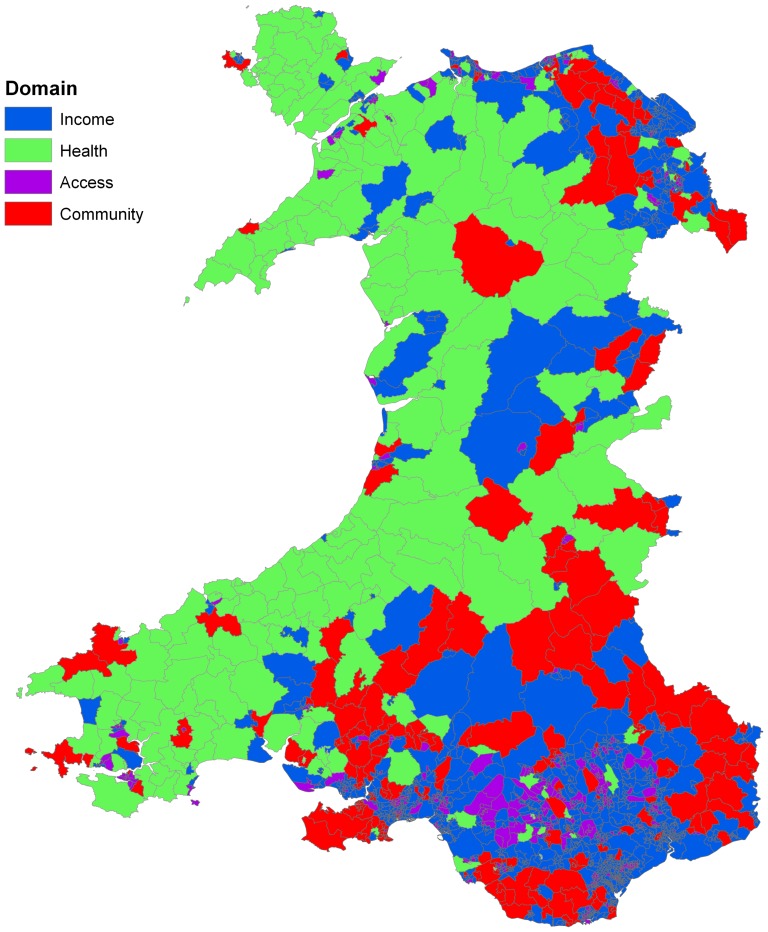
Highlighting key domains having strong association with educational performance (defined by largest MiD) across Wales.

**Figure 8:**
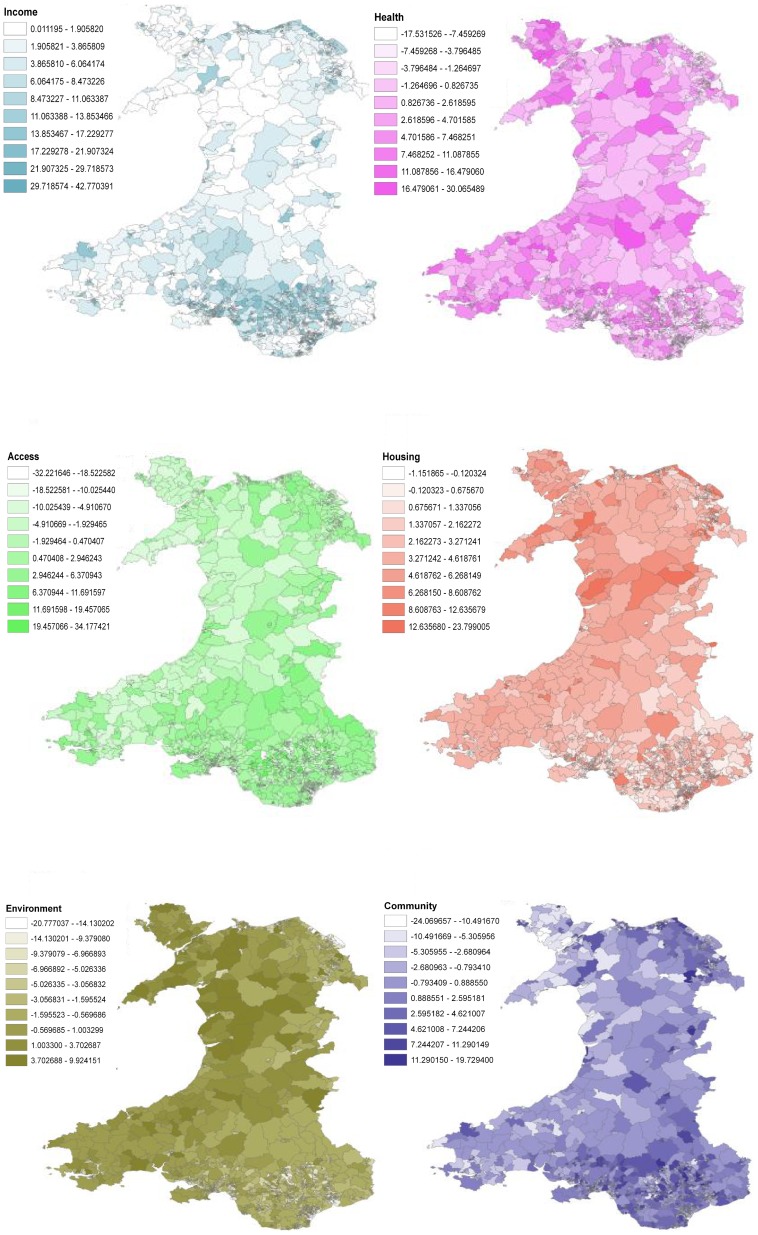
Variations in Contribution of Domain (CoD). These values represent a product of the observed WCIMD deprivation scores, and the domain coefficient scores (MiD values) in each LSOA, and represents the contribution of each type of deprivation domain to the expected education under-attainment rate in each LSOA.

Recall however, that the fitted value for each LSOA is a weighted average of all 8 rules (LLMs). Hence the need for the proposed metrics to reveal the unique local picture. The MiD values across all LSOAs are illustrated in Figure 6, in which a positive MiD indicates higher levels of deprivation are associated with higher education failure rates. Figure 7 shows the domain factor for each LSOA that has the strongest MiD across Wales. Moreover, Figure 8 illustrates the geographical trends of the CoD for each LSOA. The number of observations here are large. Based on our knowledge concerning specific LSOAs, we provide some case studies highlighting the insights from the new model and metrics.

*Case Study 1:* Affluent area, with only poor access to services. For this region, the dominant rule is *W_2_*, which suggests that in terms of the MiD metric the domain factors - *Community*, *Income* and *Health* have strong associations with educational outcomes (highest coefficients in Figure 3). However, for this area, all these domains are already at extremely low deprivation levels, making them unlikely targets for improvement. In contrast, the CoD metric shows that *Access* is overall the most important determinant of under-attainment performance (>33% contribution to the predicted educational under-attainment score), and should be the key target. This case study represents a “straightforward” situation, in which the most important target domain is simply the standout value from the WCIMD data. Here, all deprivation scores are very low, apart from *Access*. Despite the obvious main conclusion, the MiD values highlight higher than expected roles for certain domains (*Income*, *Community* and *Health*, reflected in high MiD coefficients, and moderate CoD scores).

*Case Study 2:* Area with poor access to services, and moderate levels of deprivation for other domains. This is another area for which the dominant rule is *W_2_*, and might be expected to have similar problems to the Case Study 1. It is in fact the next most deprived area in their region for *Access* (98^th^ percentile). However, despite *Access* being the standout value in the raw WCIMD data, this time a simple interpretation does not hold. Since there are moderate deprivation levels for *Health* (37^th^ percentile), and MiD coefficients show that the effect of *Health* in this region to be highest, then *Health* is classified as by far the most important target in the area.

*Case Study 3:* Deprived area, with especially poor income, health and community safety domains (but good access to services). The dominant rule is *W_6_* (also with significant weighting from *W_7_*), which implies an expected impact (according to MiD) from *Access*, *Income* and *Health*. However, due to the great disparity between high deprivation on the *Income* (94^th^ percentile) and good *Access* (39^th^ percentile), then in the CoD metric the *Income* domain (and to a lesser extent *Health*) dominates and is highlighted as the key priority.

*Case Study 4:* Area of moderate deprivation across all domains. Here, the dominant local model is *W_1_* (although with significant contribution from several other models). According to the MiD metric, *Income*, *Community* and *Housing* are expected to have the strongest relationship with educational outcome. Both metrics suggests that *Income* is the target domain, having the strongest coefficient (MiD) value and with a CoD value contributing 67% to the expected under-attainment rate.

The last two Case Studies focus on *Housing* and *Environment*. Although these factors tend to have a smaller role than the other domains, it is not difficult to identify cases in which they play important roles. *Case Study 5* represents an LSOA with generally very low deprivation (*Income*, *Health*, *Community*), but with roughly average levels of *Housing*, *Environment* and *Access*. This area is dominated by rule *W_3_* (though with significant contributions from the other rules). In terms of MiD coefficient, *Income* appears to have the strongest correlation with educational performance. However, due to the very low WCIMD values for *Income*, the largest component of under-attainment, CoD value, is *Housing*.

*Case Study 6* represents a set of rural areas, which are very affluent, yet have high deprivation scores at the *Environment* and *Access* domains. They are described predominately by rule *W_4_*, which suggests a strongest coefficient (MiD) for *Health*. However, the CoD value shows that the greatest deprivation contribution to under-attainment comes from the *Environment* domain.

We note that these examples are not easily generalized. There is a role for all aspects of deprivation, including *Environment*, *Housing*, *Access* and *Community*, in addition to the factors of *Income* and *Health* that are usually focused on. The importance in a given region is not simply revealed by the deprivation scores alone, and consequently there are very many different spatial patterns that can be found in the data, and contrasting effects found across areas that initially appear quite similar. A need for area-based interpretation is clear.

Our investigations so far suggest that, although in our modelling each LSOA is considered as independent one from another, regional characteristics (urban, rural, industrial areas, etc.) can play important roles in explaining the variations of spatial patterns. For rural areas with scattered population, such as those dominated by rule *W_2_*, *Access to Services* emerges as a key domain for improving educational performance. Areas, such as the ex-coalfields in South Wales Valleys, have the key priority in the domains of *Income* and *Health*. An extension of the approach to formally include spatial statistics would be a potential area of future research.

## Discussion

On first encounter, the algorithm for fitting the TS model can appear complicated. However it is little more than a trick for finding sub-regions of the multidimensional data space that are distinct, and can then be well described by a small set of simple models (LLMs). Then for a specific area, one uses an average of these models that reflects how well the area ‘belongs’ to the sub region of the data. Using if-then rules and local modelling technique, we suggest that the TS model offers greater transparency than fitting interactions and complex non-linear terms in, for example, regression models, because we are always dealing with a simple model in a relevant data space, rather than a complex model that tries to capture all the subtleties at once.

A statistical interaction can be defined as a relationship in which the influence of a variable on an outcome measure is dependent on the value of other (interacting) variables. In the fitting of a TS system, if there is a requirement for several LLMs this, by definition, represents interaction (or non-linearity) in the data. It means that the coefficients for a certain variable will be different (specified by a different LLM) across different regions of the data space. Hence, this approach is natural for revealing complex interactions between variables, while remaining fairly easy to interpret at the local level, where the effects are approximately linear.

As a comparison to our data mining approach, a standard linear regression analysis finds that *Access* and *Community* are not statistically significantly associated (at the 5% level) with educational attainment (in univariate analysis) and the majority of two-way interactions were not significant (see Table S3 and Table S4 of Data Supplement). Some of the complexity of the data can indeed be recovered from the presence of several significant multi-way interactions, however we argue that these models are much more difficult to interpret than the straightforward “*if-then*” linear models presented here. Nevertheless, this study has some potential limitations. First our data mining approach adopted in this study involves higher computing overhead for the purpose of constructing a parsimonious model. Moreover designing the fuzzy sets for linguistic terms needs skills and additional efforts for appropriate generation of hyper-parameter values via a trial-and-error approach.

To put some of these findings in context, the World Health Organization (WHO) has promoted the importance of early area-based interventions in health and educational attainment worldwide [34], leading to community-based initiatives to improve developmental outcomes among socio-economically disadvantaged children [35]–[37]. These initiatives reflect the WHO's principles of the Health for All (HFA) [34]. For example, the Best Start project in Australia aims to improve the overall health, development, learning and wellbeing of Victoria's young children and their families in some of the most socially disadvantaged communities through local partnerships and improved service co-ordination [38]. After several years, the findings suggest that improvements in access to services in disadvantaged areas can be achieved by area-based interventions, such as optimising the use of existing resources, and that the potential health benefits of area-based interventions might be better assessed by examining steps along the pathway between intervention and outcome [35] [39]. In the UK, improving education and skills is one of the five priority goals of the National Strategy for Neighbourhood Renewal and the New Deals for Communities Programme. This area-based approach has led to initiatives such as Sure Start, Excellence in Cities, Education Action Zones and Aim Higher which target education. The New Deals for Communities is unique in relation to previous initiatives by addressing not only education but the *other factors* that might impede progress in educational performance [37]. More specifically, it picks highly deprived neighbourhoods to tackle place based issues (crime, community, housing, physical environment) in order to address people based outcomes (education, health and worklessness) using school and community interventions. The findings of our study offer data mining evidence suggesting that a combined approach of tackling place based issues and educational attainment may be more effective than simply focusing on education alone.

In intervention evaluation, a general theme is that rigorous program evaluation is needed to determine which interventions will work most effectively and to spend scarce resources wisely [40]. However, evaluation of complex intervention is often difficult. For example, a review looking at housing improvements and health effects [41] found a large number of methodological difficulties in the before and after comparisons, especially in the response and follow-up rates among deprived communities and there is a lack of evidence on health gains that result in investment in housing. Thus, while this study suggests that tackling place and income barriers will improve children's education, there is currently a lack of evidence from intervention studies to confirm this. The scope of future evaluations must be carefully tailored and techniques must be properly selected to generate accurate information for policy makers.

## Conclusions

Complex interactions are notoriously difficult to detect and interpret within a standard statistical regression framework. This paper provided a local modelling method that relies on sets of simple linear models, and proposed two new metrics, *MiD* and *CoD*, to quantify the variations of local level impacts of multidimensional factors on educational outcomes. This study revealed some intriguing findings to bolster the scanty evidence base around the complex inter-relationship between different domains of health and socio-economic status and educational achievement. The results are consistent with a growing literature on the importance of place characteristics on individual outcomes. The findings imply that a broad range of policies may have influence in reducing inequalities in educational achievement and that interventions tailored to fit in with local characteristic would help to increase their effectiveness. It is now important to exploit the opportunities posed by natural experiments where substantial changes are made to the distribution of deprivation domains as a result of planned or serendipitous circumstances. Such longitudinal effects would serve to validate predictions of the data mining models, and aid in the assessment of causality.

## Supporting Information

Figure S1 **The prediction by the constructed TS model (circles) vs observed child educational under-attainment rates (points) at testing LSOAs.** A random sample of 50 LSOAs from the testing sample are shown here, to aid in clarity.
(DOCX)

Table S1 **Number of children with Key Stage 1 or 2 records in study, between 2005 and 2007.**
(DOCX)

Table S2 **Pair-wise linear correlation coefficients for all domains.** Note that negative association is common, and few of the domain scores are highly correlated.
(DOCX)

Table S3 **Results of linear regression applied to the same dataset.**
(DOCX)

Table S4 **Linear regression with all pairwise interactions.**
(DOCX)
