# Supplementary material for: Local Modelling Techniques for Assessing Micro-Level Impacts of Risk Factors in Complex Data: Understanding Health and Socioeconomic Inequalities in Childhood Educational Attainments
Source: PLoS One. 2014 Nov 19;9(11):e113592. doi: 10.1371/journal.pone.0113592 (PMC4237439; doi:10.1371/journal.pone.0113592)
Supplement: Figure S1 — The prediction by the constructed TS model (circles) vs observed child educational under-attainment rates (points) at testing LSOAs. A random sample of 50 LSOAs from the testing sample are shown here, to aid in clarity. (DOCX) [file pone.0113592.s001.docx]

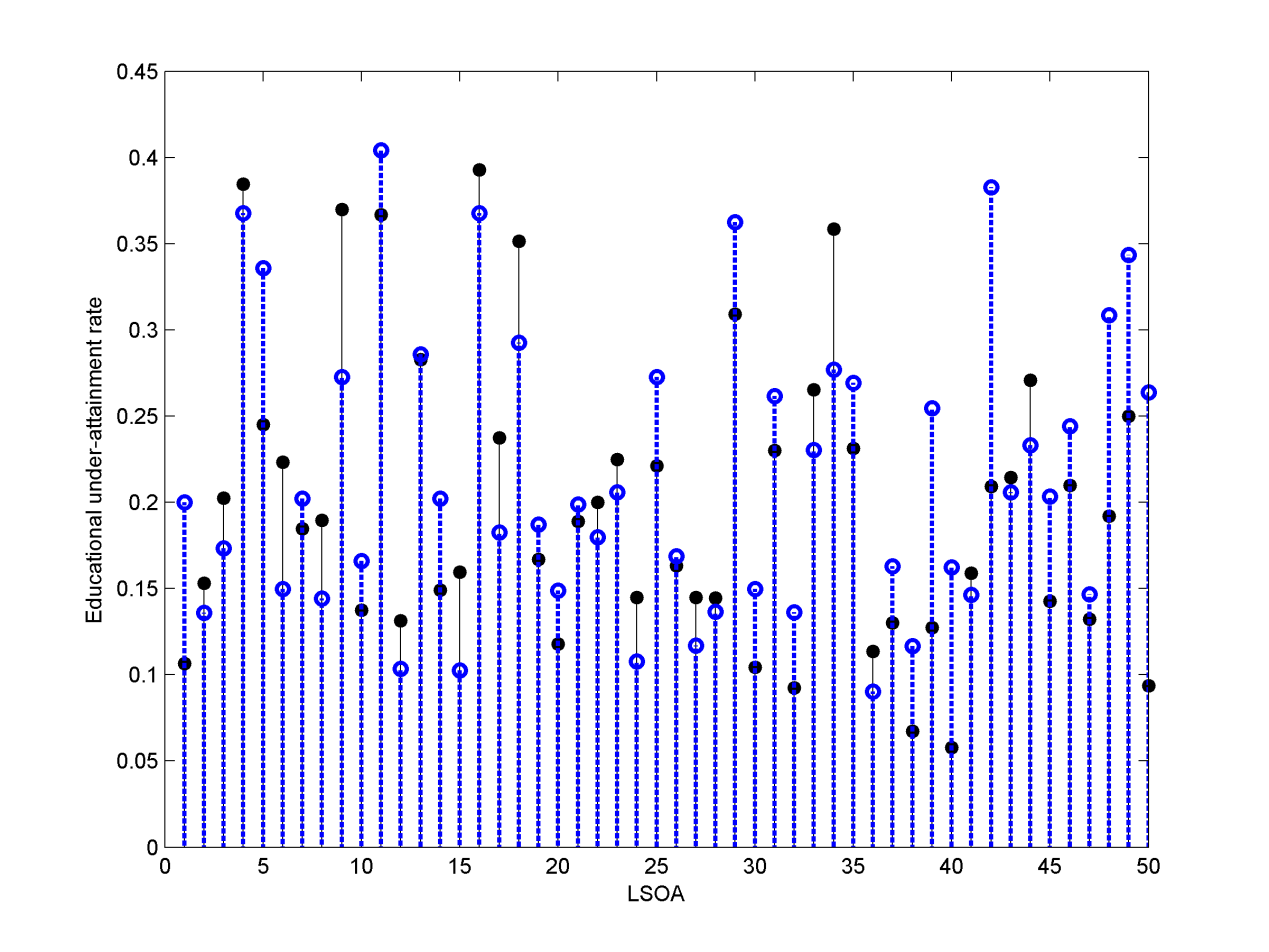


### Figure S1*.* The prediction by the constructed TS model (circles) vs observed child educational under-attainment rates (points) at testing LSOAs. A random sample of 50 LSOAs from the testing sample are shown here, to aid in clarity.
