# Supplementary material for: Local Modelling Techniques for Assessing Micro-Level Impacts of Risk Factors in Complex Data: Understanding Health and Socioeconomic Inequalities in Childhood Educational Attainments
Source: PLoS One. 2014 Nov 19;9(11):e113592. doi: 10.1371/journal.pone.0113592 (PMC4237439; doi:10.1371/journal.pone.0113592)
Supplement: Table S1 — Number of children with Key Stage 1 or 2 records in study, between 2005 and 2007. (DOCX) [file pone.0113592.s002.docx]

### *Table S1.* Number of children with Key Stage 1 or 2 records in study, between 2005 and 2007

|  | ***Number of child records*** | ***Number of records showing under-attainment*** |
| --- | --- | --- |
| KS1 | 94960 | 18307 |
| KS2 | 101810 | 26307 |
| Total | 196770 | 44614 |
