# Supplementary material for: Local Modelling Techniques for Assessing Micro-Level Impacts of Risk Factors in Complex Data: Understanding Health and Socioeconomic Inequalities in Childhood Educational Attainments
Source: PLoS One. 2014 Nov 19;9(11):e113592. doi: 10.1371/journal.pone.0113592 (PMC4237439; doi:10.1371/journal.pone.0113592)
Supplement: Table S2 — Pair-wise linear correlation coefficients for all domains. Note that negative association is common, and few of the domain scores are highly correlated. (DOCX) [file pone.0113592.s003.docx]

### *Table S2:* Pair-wise linear correlation coefficients for all domains. Note that negative association is common, and few of the domain scores are highly correlated.

|  | Income | Health | Access | Housing | Environment | Community |
| --- | --- | --- | --- | --- | --- | --- |
| Income | 1 | 0.669614 | -0.29511 | 0.421748 | 0.134871 | 0.761586 |
| Health | 0.669614 | 1 | -0.27937 | 0.277877 | 0.078824 | 0.564551 |
| Access | -0.29511 | -0.27937 | 1 | -0.03869 | -0.19662 | -0.43837 |
| Housing | 0.421748 | 0.277877 | -0.03869 | 1 | 0.08626 | 0.43071 |
| Environment | 0.134871 | 0.078824 | -0.19662 | 0.08626 | 1 | 0.196722 |
| Community | 0.761586 | 0.564551 | -0.43837 | 0.43071 | 0.196722 | 1 |
