# Supplementary material for: Local Modelling Techniques for Assessing Micro-Level Impacts of Risk Factors in Complex Data: Understanding Health and Socioeconomic Inequalities in Childhood Educational Attainments
Source: PLoS One. 2014 Nov 19;9(11):e113592. doi: 10.1371/journal.pone.0113592 (PMC4237439; doi:10.1371/journal.pone.0113592)
Supplement: Table S3 — Results of linear regression applied to the same dataset. (DOCX) [file pone.0113592.s004.docx]

### *Table S3.* Results of linear regression applied to the same dataset^*^

| ***Independent***  ***Variable*** | ***100 ×*** ***Coefficient*** | ***Standard Error*** | ***t-statistic*** | ***p-value*** |
| --- | --- | --- | --- | --- |
| Constant | 13.459 | 0.0039 | 34.270 | .000 |
| Income Deprivation | 0.308 | 0.0001 | 23.684 | .000 |
| Health Deprivation | 0.047 | 0.0001 | 4.626 | .000 |
| Access Deprivation | -0.001 | 0.0001 | -.133 | .894 |
| Housing Deprivation | 0.046 | 0.0001 | 5.467 | .000 |
| Environment Deprivation | -0.016 | 0.0001 | -2.143 | .032 |
| Community Deprivation | 0.006 | 0.0001 | .487 | .626 |

^*^Note: In simple univariate analyses, *Access* and *Community* are not significantly related to education under-attainment rate.
