# Supplementary material for: Local Modelling Techniques for Assessing Micro-Level Impacts of Risk Factors in Complex Data: Understanding Health and Socioeconomic Inequalities in Childhood Educational Attainments
Source: PLoS One. 2014 Nov 19;9(11):e113592. doi: 10.1371/journal.pone.0113592 (PMC4237439; doi:10.1371/journal.pone.0113592)
Supplement: Table S4 — Linear regression with all pairwise interactions. (DOCX) [file pone.0113592.s005.docx]

### *Table S4.* Linear regression with all pairwise interactions^**^

| ***Independent Variable*** | ***100 × Coefficients*** | ***Standard Error*** | ***t-statistic*** | ***p-value*** |
| --- | --- | --- | --- | --- |
| Constant | 10.597 | .633 | 16.728 | .000 |
| Income | 0.510 | .037 | 13.933 | .000 |
| Health | 0.085 | .027 | 3.122 | .002 |
| Access | 0.004 | .017 | .263 | .793 |
| Housing | 0.064 | .024 | 2.722 | .007 |
| Environment | 0.026 | .019 | 1.394 | .163 |
| Community | -0.040 | .032 | -1.260 | .208 |
| IncomeHealth | -0.002 | .001 | -3.648 | .000 |
| IncomeAccess | 0.000 | .001 | .289 | .772 |
| IncomeHousing | -0.003 | .001 | -4.297 | .000 |
| IncomeEnv | -0.002 | .001 | -2.566 | .010 |
| IncomeComm | -0.001 | .001 | -2.379 | .017 |
| HealthAccess | -0.001 | .001 | -1.058 | .290 |
| HealthHousing | 0.000 | .001 | -.727 | .467 |
| HealthEnv | -0.000 | .000 | -.204 | .838 |
| HealthComm | 0.001 | .001 | 1.920 | .055 |
| AccessHousing | 0.000 | .000 | .148 | .883 |
| AccessEnv | -0.001 | .001 | -1.399 | .162 |
| AccessComm | 0.003 | .001 | 3.161 | .002 |
| HousingEnv | 0.001 | .000 | 2.488 | .013 |
| HousingComm | 0.001 | .001 | 2.187 | .029 |
| EnvComm | 0.000 | .001 | -.402 | .688 |

^**^Note: The complexity of the data starts to be revealed by the interaction terms. In the TS model approach, these interactions are represented by linear models in distinct sub-regions of the multi-dimensional deprivation score space. Note that multi-way interactions were also found to be significant, however at this point the interpretability of the standard regression approach becomes very complicated.
